# Relationship Between Heated Tobacco Products and Placental Abruption: A Prospective Cohort Study Using Online Questionnaire

**DOI:** 10.1007/s44197-025-00373-2

**Published:** 2025-02-20

**Authors:** Hikaru Ooba, Jota Maki, Takahiro Tabuchi, Hisashi Masuyama

**Affiliations:** 1 Center for Innovative Clinical Medicine, Okayama University Hospital, Okayama, Japan; 2 Department of Obstetrics and Gynecology, Okayama University Graduate School of Medicine, Dentistry and Pharmaceutical Sciences, 2-5-1 Shikata-cho, Kita-Ku, Okayama, Okayama 700-8558 Japan; 3 Division of Epidemiology, School of Public Health, Tohoku University Graduate School of Medicine, Sendai, Japan

**Keywords:** Placental abruption, Heated tobacco products, Pregnancy, Tobacco use

## Abstract

**Background:**

Placental abruption (PA) is a critical obstetric complication, with maternal smoking recognized as a key risk factor. Despite the increased use of heated tobacco products (HTPs), the impact of HTPs remains unclear. This study investigated whether pregnant women using HTPs are at a higher risk of PA than non-users.

**Methods:**

We analyzed data from “the Japan COVID-19 and Society Internet Survey,” a prospective, self-reported online survey cohort. Questionnaires were randomly distributed between July 28, 2021, and August 30, 2021. Pregnant respondents in 2021 were invited to complete an additional survey from February 14, 2022, to February 28, 2022. We set the outcome as the absolute risk difference (aRD) and relative risk ratio (rRR) of PA incidence due to smoking HTPs in the first trimester of pregnancy. The sample size included 12 836 participants. We calculated outcomes using a generalized linear model (GLM) and inverse probability of treatment weighting (IPTW). We also performed a Bayesian approach and multiple-bias analysis for sensitivity analysis.

**Results:**

We found the robust aRD of 0.07 (95% confidence interval (CI): 0.06, 0.09) and the rRR of 11.3 (95% CI: 7.5, 17.0). Multiple bias analyses showed that unmeasurable confounders would need to have at least an rRR = 14 relationship with both exposure and outcome to disprove the observed association. There has not been post hoc analysis or secondary use of data.

**Conclusion:**

Early pregnancy use of HTPs is associated with an increased risk of PA.

## Introduction

Placental abruption (PA) is a critical obstetric complication characterized by the separation of the placenta from the uterine wall during pregnancy or before the completion of labor [1]. This condition increases mortality and the likelihood of severe complications for both the mother and neonate [2]. Among several factors contributing to the incidence of PA, maternal smoking has been identified as the primary risk factor [3].

Recently, the use of heated tobacco products (HTPs) has increased. HTPs are electronic devices that heat tobacco leaves to generate aerosols [4]. In advertising campaigns, HTPs tend to be considered less harmful alternatives to combustible tobacco [5]. However, HTPs are still associated with undesirable health effects, such as cardiovascular diseases. Cross-sectional studies have revealed that the use of HTPs may increase the risk of hypertensive disorders of pregnancy [6]. Consequently, HTPs have the potential to cause pregnancy-related complications associated with smoking.

The association between exposure to HTP aerosols and PA remains insufficiently elucidated owing to the low prevalence of smoking among pregnant women and the rarity of PA incidence. Previous studies indicate that the rate of HTP usage in the general population is 12.6% [6], and the incidence rate of PA in the pregnant population ranges from 0.4 to 1.0% of all pregnancies [7]. In such cases, obtaining large, high-quality samples at appropriate times is difficult. However, neglecting to conduct research due to insufficient statistical power might result in ignoring significant maternal and child health risk factors. This survey-based study aimed to investigate whether the risk of PA in pregnant women who use HTPs is higher than that in those who do not.

## Methods

### Study Setting and Population

We analyzed participant data from the Japan COVID-19 and Society Internet Survey [8]. This prospective, self-reported, online survey was conducted during the COVID-19 pandemic to investigate changes in lifestyle, health, social life, and economic activities, including the smoking status of HTPs. To prevent missing responses, the questionnaire was designed to progress to the next question only after answering the current question. Between July 28, 2021, and August 30, 2021, questionnaires were randomly distributed to 440 323 participants, stratified by sex, age, and prefecture, representing the entire Japanese population. An original algorithm was used to exclude illogical or inconsistent responses. Of the remaining participants, those who reported being pregnant in the 2021 survey were subjected to an additional online survey conducted from February 14, 2022, to February 28, 2022. Excluding fraudulent responders and those still pregnant at the time of this follow-up survey, the remaining participants were considered for analysis. To enhance willingness to participate, respondents were offered credit points.

### Measurement

We investigated the smoking status of HTPs during the first trimester of pregnancy (up to 13 weeks and 0 days). HTPs were defined as products of the Ploom^®^, IQOS^®^, and glo^®^ series. Participants self-reported the approximate number of HTPs smoked per day during this period. Based on these self-reports, a binary variable was created where “0” represented ‘non-smoking’ and “1” indicated ‘smoking’.

The primary outcome was defined as the absolute risk difference (aRD) of PA incidence due to smoking HTPs during the first trimester of pregnancy. The secondary outcome was the relative risk ratio (rRR) of PA incidence associated with HTP smoking during the same period. Participants were requested to self-report occurrences of PA based on entries in their Maternal and Child Health Handbook. In Japan, this handbook is distributed to all pregnant women and meticulously records medical visits before and after pregnancy at various healthcare facilities [9]. The diagnosis of PA that was expected to be documented in the handbook was made by their physician.

The sample size [10] was conducted as follows: assuming an exposure rate of 12.6%, an average incidence rate of placental abruption (PA) in pregnant women of 0.7%, an aRD of 0.7%, a significance level of 5%, and a power of 80%. We also assumed 30% dropout rate for the initial questionnaire, 20% response rate for identifying pregnant women, and 30% dropout rate for the follow-up questionnaire. Based on these assumptions, the sample size was calculated as 12 836 participants. Considering the various risk factors involved in the incidence of PA [7], the following covariates were selected: age ≥ 35 or ≤ 20 years; multiparity ≥ 3; in vitro fertilization; alcohol use during pregnancy; hypertensive disorders of pregnancy; pre-eclampsia; gestational diabetes; thyroid dysfunction; previous history of PA; premature rupture of membranes; placenta previa; low gestation period; fetal sex; traditional tobacco smoking during the first trimester; partner’s HTP smoking; and partner’s traditional tobacco smoking. The term ‘traditional tobacco’ includes rolled cigars, small cigars, and cigarettes. Smoking status was categorized as a binary variable using the same criteria as HTPs.

### Statistical Analysis

Based on the 2021 baseline responses, we presented the characteristics of the pregnant respondents, including covariates. Subsequently, the responses from 2021 to 2022 were linked using a common respondent ID.

As PA can occur due to several factors, we adjusted for biases by calculating propensity scores (PS) using measured covariates and conducting inverse probability of treatment weighting (IPTW). The IPTW method weights the exposed group by the inverse of the probability of being exposed and the control group by the inverse of the probability of being a control. Under certain assumptions, this method can estimate the average treatment effect (ATE) across the entire population [11]. To select the variables to include in the PS, we adopted the Disjunctive Cause Criterion [12]. This criterion, which aims to balance covariates, assumes that controlling for each covariate that is a cause of exposure, outcome, or both will achieve confounding control [13]. Variables known to be instrumental were excluded from this set. Proxies for unmeasured variables that were common causes of both the exposure and the outcome were included as covariates. Conditions such as hypertensive disorders of pregnancy, premature rupture of membranes, and low birth weight were considered mediators between smoking and PA, and adjusting for these factors could result in over-adjustment [14]. Therefore, we excluded these variables. PS was calculated based on logistic regression with covariates as explanatory variables and HTPs smoking status as the dependent variable.

We calculated aRD using a generalized linear model (GLM) with a Gaussian distribution and an identity link function [15], along with standard errors estimated by the sandwich estimator. For rRR, we employed a modified Poisson regression model [16]. This GLM model uses a Poisson distribution and logarithmic link function, with standard errors calculated using the sandwich estimator. While applying the regression model to binary data may overestimate the estimated standard error, the sandwich estimator procedure provides robust estimates. A 95% confidence interval (CI) was calculated for each estimate.

### Sensitivity Analyses

With the small number of individuals who smoke HTPs and develop PA, concerns have arisen regarding the instability of risk estimates. Therefore, we adopted a Bayesian approach for the sensitivity analysis. IPTW scales the population into a comparable pseudo-population that cannot be directly integrated into the data-generating process of Bayesian models [17]. To address this limitation, an approach incorporating Bayesian estimation has been proposed [18]. This approach was divided into a two-step process. First, Bayesian models were used to estimate the likelihood of exposure and generate posterior distributions of PS. Based on this, PS samples were generated. Then, IPTW was produced for each sample, and these weights were utilized in running the outcome model. The results of the models were averaged to calculate the final ATE.

Based on previous studies [6, 7], we assumed that the average smoking rate of HTPs was 12.6% and the average incidence rate of PA was 0.7%. The Cauchy distribution with a scale of 2.5 is known for its heavy tails, making it robust for prior distribution in situations with outliers or high uncertainty [19]. Therefore, we set the prior distribution for the prevalence of HTP smoking and PA using the Cauchy distribution. No prior information was available to estimate PS distribution. In such situations, uniform or standard normal distributions serve as uninformative prior distributions. Hence, we selected a uniform uninformative prior distribution between − 10 and 10. We utilized the No-U-Turn Sampler (NUTS), an extension of the Hamiltonian Monte Carlo method, for sampling from the posterior distribution [20]. During the sampling process, 10,000 iterations were performed. We also calculated the highest density interval (HDI). The Bayesian model was implemented using the PyMC library version 5.0 [21].

In this study, detailed data on variables such as race and medication history were not available. To adjust for possible confounders, we conducted non-probabilistic multiple bias analyses with bounding factor [22]. This analysis was adjusted for potential biases induced by unmeasured confounding factors (known as boundary factors [23]) to assess the robustness of the rRR estimates. Let RR_UE_ be the rRR of the impact of unmeasurable confounding factors on HTP smoking, RR_UD_ be the impact on PA incidence, B be the boundary factor, RR_PRE_ be the pre-adjustment rRR, and RR_POST_ be the post-adjustment rRR. The boundary factor was calculated using the following formula (1):

$$\:B=\frac{R{R}_{\text{U}\text{D}}\times\:R{R}_{\text{U}\text{E}}}{R{R}_{\text{U}\text{D}}+R{R}_{\text{U}\text{E}}-1}\:\:\left(1\right)$$

Using this Bounding factor, RR_POST_ was calculated using the following formula (2):

$$\:R{R}_{\text{P}\text{O}\text{S}\text{T}}=\frac{R{R}_{\text{P}\text{R}\text{E}}}{B}\:\:\left(2\right)$$

We plotted the calculated RR_POST_ values while varying the magnitudes of RR_UE_ and RR_UD_ from 1 (no association) to 15 (strong association). All figures were generated using Matplotlib version 3.7.1 (Matplotlib Development Team, Portland, OR, USA) and Plotly version 5.15.0 (Plotly Technologies Inc., Montreal, QC, Canada). All analyses were conducted using Python version 3.10.12 (Python Software Foundation, Wilmington, DE, USA).

## Results

**Fig. 1:**
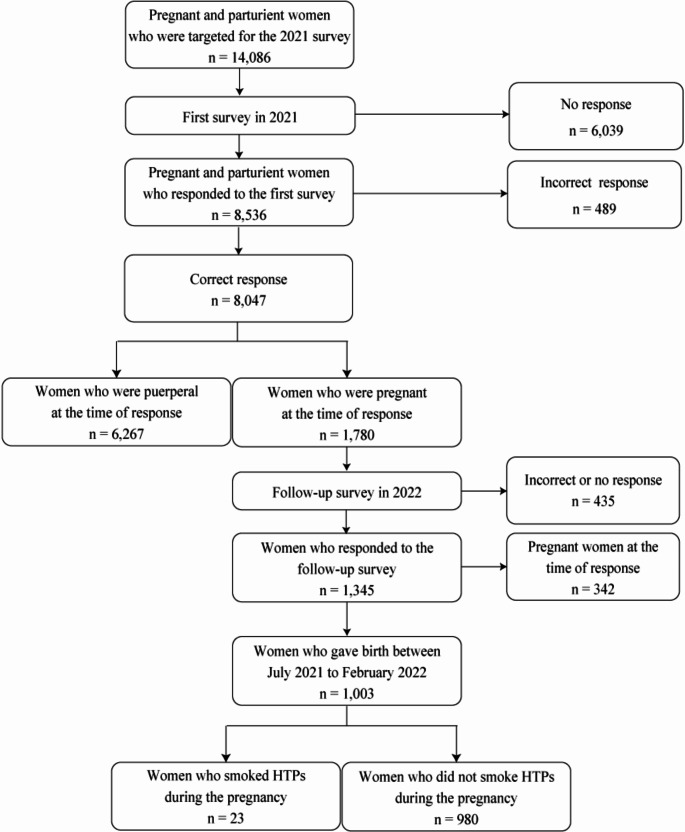
Recruiting flow of the target population

In 2021, questionnaires were sent to 14 086 individuals, receiving 8536 responses (60.6%). However, 489 responses (3.5%) were fraudulent and were excluded, leaving 8047 (57.1%) participants eligible. Of these, 1780 (22.1%) were pregnant at the time of their response. In the 2022 follow-up survey, 435 individuals (3.1%) were either unreachable or deemed fraudulent and were excluded, resulting in 1345 (9.5%) responses. From the 2022 participants, we excluded 342 (2.4%) who were still pregnant, finalizing 1003 (7.1%) subjects for analysis (Fig. 1). No multiple imputation of data was necessary, as there were no missing values in the analysis factors.

**Fig. 2:**
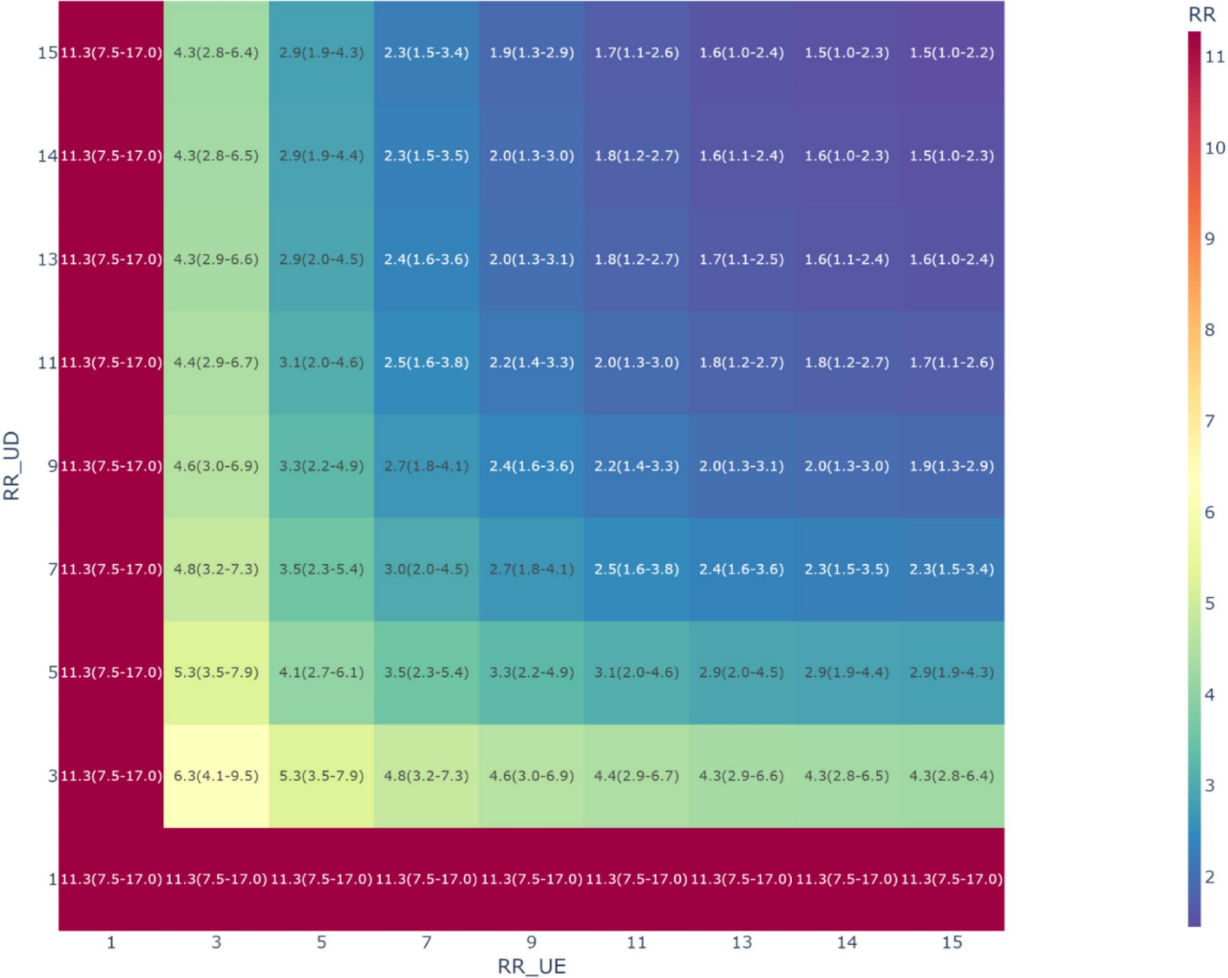
Association between the smoking of heated tobacco products (HTPs) and elevated occurrence of placental abruption (PA) after adjusting for unmeasured confounders. RR, observed risk ratio between HTPs and PA; RR_UE_, the risk ratio of unmeasured confounders on smoking HTPs; RR_UD_, the risk ratio of unmeasured confounders on PA

Without adjustment, we found the aRD of 0.08 (95% CI: 0.04, 0.12) and the rRR of 13.2 (95% CI: 2.6, 67.6). Under IPTW, we found the robust aRD of 0.07 (95% CI: 0.06, 0.09) and the rRR of 11.3 (95% CI: 7.5, 17.0). The c-statistic for the propensity score was 0.82. Applying Bayesian IPTW, the aRD was 0.08 (94% HDI: 0.06, 0.11), and the rRR was 11.9 (94% HDI: 4.6, 30.2). In all Bayesian estimations, the $\:\widehat{r}$ values were below 1.01, indicating convergence. Our multiple bias analyses indicated that unmeasurable confounders would require a minimum RR_UE_ and RR_UD_ of 14, with both exposure and outcome, to nullify the observed association. (Fig. 2), (Table. 1).

**Table 1:** Background of participants. HTPs: heated tobacco products. *: *p* < 0.05, **: *p* < 0.01

		Smoking HTPs		*p*-value
		(−) (*n* = 980)	(+) (*n* = 23)	
Maternal age ≥ 35 years or < 20 years	(−)	721	18	0.79
	(+)	259	5	
Parity ≥ 3	(−)	951	23	0.84
	(+)	29	0	
In vitro fertilization	(−)	966	20	< 0.001**
	(+)	14	3	
Drinking alcohol	(−)	942	23	0.68
	(+)	38	0	
Pregestational chronic hypertension	(−)	975	22	0.32
	(+)	5	1	
Pregestational diabetes mellitus	(−)	976	22	0.25
	(+)	4	1	
Pregestational thyroid dysfunction	(−)	945	21	0.47
	(+)	35	2	
Previous history of placental abruption	(−)	979	22	0.03*
	(+)	1	1	
Hypertensive disorders of pregnancy	(−)	922	22	1.0
	(+)	58	1	
Premature rupture of membranes	(−)	894	18	0.08
	(+)	86	5	
Placenta previa	(−)	955	21	0.25
	(+)	25	2	
Small for gestational age fetus	(−)	912	21	1.0
	(+)	68	2	
Male fetal sex	(−)	487	9	0.43
	(+)	493	14	
Smoking traditional tobacco in the first trimester of pregnancy	(−)	966	20	< 0.001**
	(+)	14	3	
smoking HTPs within 3 months immediately before pregnancy	(−)	958	2	< 0.001**
	(+)	22	21	
Partner’s smoking of traditional tobacco	(−)	855	15	0.005**
	(+)	125	8	
Partner’s smoking of HTPs	(−)	830	10	< 0.001**
	(+)	150	13	
Placental abruption during this pregnancy	(−)	973	21	0.004**
	(+)	7	2	

## Discussion

This study revealed that smoking HTPs during early pregnancy is a risk factor for PA. This trend persisted even after adjusting for measurable risk factors. The multiple bias analyses we conducted revealed that to deny the observed association, unmeasurable confounders would require a relationship of at least RR_UE_=14 and RR_UD_=14 with both exposure and outcome. Typically, a small number of events can weaken the statistical power and lead to extreme parameter estimations [24]. In such cases, Bayesian estimation using information-rich prior distributions can reduce the impact of variance [25]. The similarity between estimations based on frequentist statistics and Bayesian statistics suggested that our results were robust.

Previous studies have suggested that nicotine is responsible for the relationship between PA and smoking. Nicotine is a pharmacologically active compound found in tobacco smoke. It rapidly crosses the fetal-placental barrier [26] and accumulates in the fetus at concentrations 15% higher than those in the mother [27]. Smoking HTPs during early pregnancy is associated with thickening of the chorionic villous basement membrane and a reduction in angiogenesis. Additionally, the vasoconstrictive effects of nicotine and hypoxia due to carbon monoxide have been implicated in causing microthrombosis in placental vessels, potentially leading to placental abruption [24]. Although HTPs contain less nicotine than traditional cigarettes [28], reduced exposure to harmful substances in tobacco does not mean a reduced risk [29]. Our observations support this hypothesis.

This study had several limitations. First, there was a potential for attrition bias [30] due to follow-up loss. For example, individuals who smoked during early pregnancy might have forgotten to respond to the follow-up questionnaire or could not be tracked because of miscarriage. Second, the observed effects were evaluated in terms of ATE, which might have led to heterogeneity in weighting across subsets of the sample. Third, the dose-dependency of PA incidence on the number of HTPs smoked remains unclear. Lastly, as this was predominantly a questionnaire-based study involving Japanese participants, further research involving different populations is warranted.

In conclusion, smoking HTPs during early pregnancy was associated with an increased risk of PA. Bias analysis provided quantitative information on the potential magnitude of bias due to unmeasured confounding variables.

## Data Availability

The data underlying this article will be shared upon reasonable request with the corresponding author.

## Abbreviations

PA: Placental abruption
HTPs: Heated tobacco products
aRD: Absolute risk difference
rRR: Relative risk ratio
GLM: Generalized linear model
PS: Propensity score
IPTW: Inverse probability of treatment weighting
ATE: Average treatment effect
95% CI: 95% confidence interval
94% HDI: 94% Highest density interval
